# Pseudo-Pneumothorax Due to Skin Folds in a Patient With Pneumonia

**DOI:** 10.7759/cureus.37564

**Published:** 2023-04-14

**Authors:** Basil Jalamneh, Ameed Taher, Ismael Nassar, Tariq Musleh, Bashar Shamieh

**Affiliations:** 1 Faculty of Health Sciences, An-Najah National University, Nablus, PSE; 2 Department of Medicine, Jenin Government Hospital, Palestinian Ministry of Health, Jenin, PSE; 3 Department of Radiology, Al-Essra Hospital, Amman, JOR; 4 Department of Emergency, Saint Joseph Hospital, Jerusalem, PSE; 5 Department of Radiology, Saint Joseph Hospital, Jerusalem, PSE

**Keywords:** tube thoracostomy, chest radiography, skin folds, pneumothorax, pseudo-pneumothorax

## Abstract

Pseudo-pneumothorax refers to several conditions that can mimic pneumothorax on chest radiography, leading to diagnostic uncertainty and unnecessary interventions. These include skin folds, bed sheet folds, clothes, scapular borders, pleural cysts, and elevated hemidiaphragm. We report a case of a 64-year-old patient with pneumonia whose chest radiograph revealed, in addition to the typical pneumonia findings, what appeared similar to bilateral pleural lines raising the suspicion of bilateral pneumothorax, but this finding was not supported clinically. Careful reexamination and further imaging ruled out the possibility of pneumothorax and concluded that this was the result of artifacts produced by skin folds. The patient was admitted and received intravenous antibiotics and was discharged three days later in stable condition. Our case highlights the importance of careful examination of imaging findings before unnecessarily proceeding to tube thoracostomy, especially when the clinical suspicion of pneumothorax is low.

## Introduction

Pneumothorax is the collapse of the lung when air accumulates inside the pleural space, which can happen spontaneously or as a result of trauma. Spontaneous pneumothorax is further classified as primary (absence of an underlying lung disease) or secondary (presence of an underlying lung disease, such as chronic obstructive lung disease and pneumonia) [1,2]. Pneumothorax is a potentially life-threatening condition that needs early recognition and prompt intervention. Several conditions, however, can mimic pneumothorax (pseudo-pneumothorax) in terms of radiologic appearance, potentially leading to unnecessary interventions. Examples include skin folds, bed sheet folds, and pleural cysts [3-5].

Here, we present a case of a patient diagnosed with pneumonia whose chest radiograph showed radiopaque lines similar to the pleural lines indicative of pneumothorax, but suspicions were eventually cleared upon further examination and imaging, as these lines turned out to be mere artifacts produced by skin folds.

## Case presentation

A 64-year-old woman with a history of congestive heart failure, diabetes, and hypertension presented to the emergency department complaining of a cough productive of yellowish sputum accompanied by shortness of breath that she had been experiencing for four days despite taking roxithromycin as prescribed by her family physician. The patient denied having experienced any chest pain or fever during this period, and she had no smoking history. She was in mild respiratory distress upon inspection, and her chest examination revealed good air entry bilaterally with mild bibasilar crackles and mild diffuse wheezes. She was afebrile and her oxygen saturation was 97%. Blood testing demonstrated a white blood cell (WBC) count of 14,900/µL and a C-reactive protein (CRP) level of 103 mg/L.

**Figure 1:**
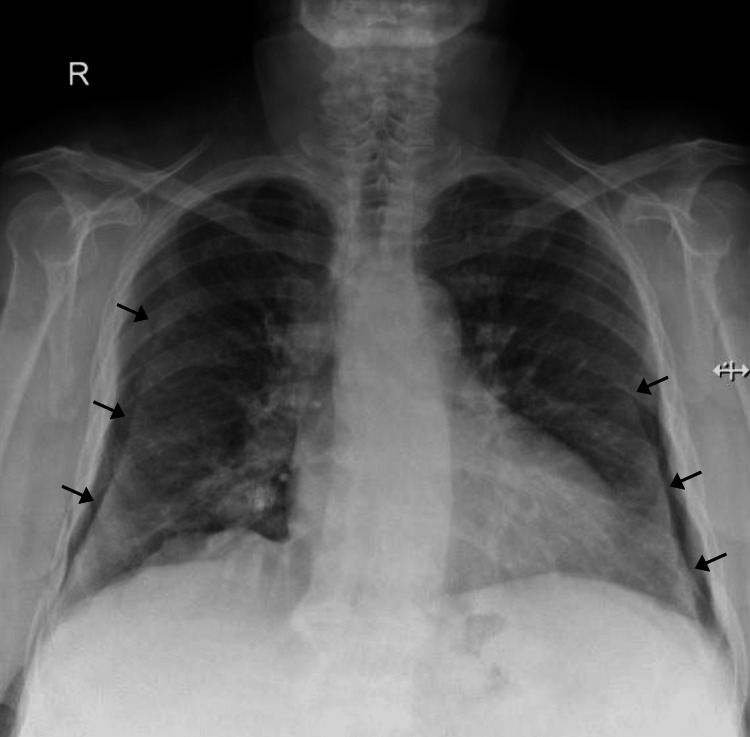
Postero-anterior (PA) chest X-ray showing bilateral curvilinear lines similar to the pleural lines seen in pneumothorax (black arrows).

A chest X-ray was obtained and showed an enlarged cardiac shadow along with infiltrates in the middle zone of the right lung, which was compatible with pneumonia. However, there were also bilateral radiopaque lines that appeared similar to the pleural lines seen in pneumothorax (Figure 1).

**Figure 2:**
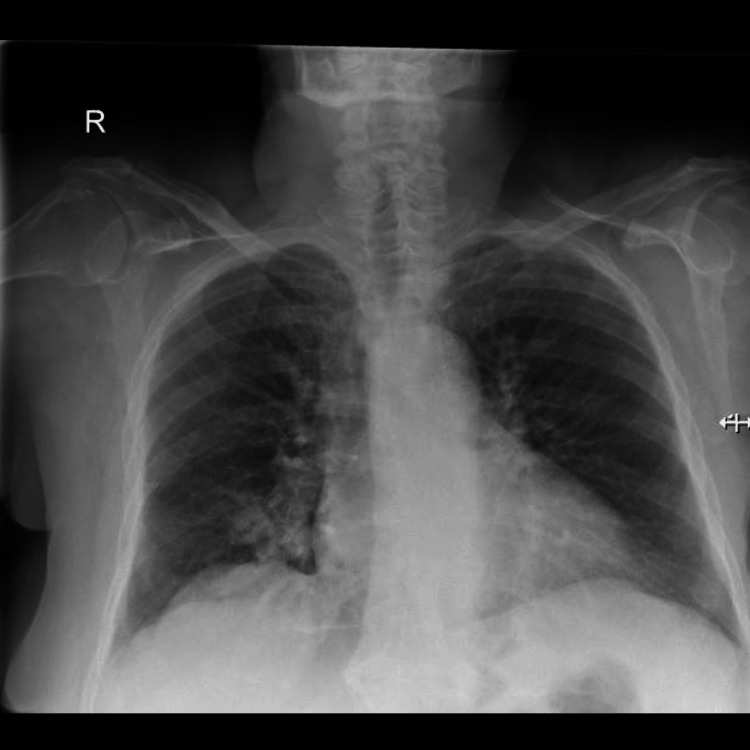
The repeated PA chest X-ray where the curvilinear lines are no longer apparent, ruling out the presence of pneumothorax. PA: postero-anterior

This prompted the physician to reexamine the patient and reevaluate the lung auscultatory findings, but the examination revealed again good air entry bilaterally, and the patient's vital signs were stable. The physician contemplated the immediate placement of a tube thoracostomy to prevent potential further deterioration, but because his suspicion of bilateral pneumothorax was not supported clinically, he decided to obtain a second chest radiograph, hoping it could clarify the disparity between clinical and radiologic findings. The repeated radiograph showed no signs of pneumothorax, as the radiopaque lines were no longer apparent (Figure 2), and so those two lines in the first radiograph were considered to be artifacts produced by skin folds.

The patient was then admitted and treated for pneumonia with intravenous levofloxacin. Her clinical condition improved by the next day, and the WBC count dropped to 11,600/µL and the CRP level became 11.6 mg/L. She was discharged two days later in stable condition after she was switched to an oral antibiotic regimen.

## Discussion

Several conditions have been reported to potentially give the false appearance of a pneumothorax on plain chest radiographs, and these have been collectively referred to as "pseudo-pneumothorax". They include skin folds, bed sheet folds, clothes, pleural cysts, medial border of the scapula, and the elevated hemidiaphragm [3-6]. A skin fold in the chest wall can resemble pneumothorax by producing a curvilinear shadow over the lung that is similar to the visceral pleural line produced by pneumothorax when air accumulates inside the pleural space separating the visceral from the parietal pleura.

Nevertheless, some radiologic differences between pneumothorax and pseudo-pneumothorax can help distinguish the two in most cases. For one, lung markings may be seen extending beyond the skin fold shadow (as was in our case), but they do not typically extend beyond the visceral pleural line in pneumothorax. An increase in lucency should also be expected lateral to the pleural lines in pneumothorax due to the presence of air, but this finding can be subtle in small pneumothorax. Another difference is that the skin fold shadow is not expected to follow the typical course of a pleural line and might even extend beyond the confines of the pleural space or end abruptly. Skin fold shadow also tends to be broader when compared with the sharp and distinct pleural line of pneumothorax [7]. The radiograph findings should also correspond with the patient's clinical picture. Our patient had no chest pain, her vital signs were stable, and her chest examination revealed good air entry bilaterally, so a pneumothorax diagnosis was unlikely on clinical grounds.

Repeating chest radiography can solve the issue if one is still in doubt, but it is still possible that the artifacts are going to appear once again on the second radiograph in a similar fashion. Other possible solutions are ultrasonography and computed tomography (CT) of the chest. Although a CT scan remains the gold standard in detecting pneumothorax, ultrasonography can serve as a viable alternative. Ultrasonography is quicker to perform, more cost-effective, and eliminates radiation exposure [8]. However, its main downside is the dependence of its accuracy on the skill of the operator [9].

Distinguishing between pneumothorax and pseudo-pneumothorax is critical, as unnecessary interventions can lead to serious complications.Niazi et al. reported a case of a patient with pseudo-pneumothorax due to a skin fold that was misdiagnosed as pneumothorax and therefore underwent a tube thoracostomy, which led to vascular injury and cardiac arrest [10]. Other possible tube thoracostomy complications include nerve damage, fistula formation, infections, and organ perforations [11]. The incidence of pseudo-pneumothorax cases that led to unnecessary interventions is not known, but it has been suggested that such occurrences are probably under-reported [12]. It is, therefore, crucial that physicians should carefully examine the imaging findings before proceeding to tube thoracostomy, especially when the clinical suspicion of pneumothorax is low.

## Conclusions

The case presented emphasizes the methods of distinguishing between pneumothorax and the pseudo-pneumothorax on radiographs to exclude the possibility of false positive results. This case is primarily educational in nature, and its main goal is to raise the issue of pseudo-pneumothorax among the medical community in an effort to minimize unnecessary invasive interventions that could occur if clinicians missed the idea of pseudo-pneumothorax.
